# Switchable graphene-substrate coupling through formation/dissolution of an intercalated Ni-carbide layer

**DOI:** 10.1038/srep19734

**Published:** 2016-01-25

**Authors:** Cristina Africh, Cinzia Cepek, Laerte L. Patera, Giovanni Zamborlini, Pietro Genoni, Tevfik O. Menteş, Alessandro Sala, Andrea Locatelli, Giovanni Comelli

**Affiliations:** 1 IOM-CNR Laboratorio TASC, Area Science Park, s.s. 14 km 163.5, Basovizza, 34149 Trieste, Italy; 2 Department of Physics, Università degli Studi di Trieste, via Alfonso Valerio 2, 34127 Trieste, Italy; 3 Peter Grünberg Institute (PGI-6), Research Center Jülich, 52425 Jülich, Germany; 4 Department of Physics, Università degli Studi di Milano, Via Celoria 16, 20133 Milano, Italy; 5 Elettra-Sincrotrone Trieste S.C.p.A., s.s. 14 km 163.5, Basovizza, 34149 Trieste, Italy

## Abstract

Control over the film-substrate interaction is key to the exploitation of graphene’s unique electronic properties. Typically, a buffer layer is irreversibly intercalated “from above” to ensure decoupling. For graphene/Ni(111) we instead tune the film interaction “from below”. By temperature controlling the formation/dissolution of a carbide layer under rotated graphene domains, we reversibly switch graphene’s electronic structure from semi-metallic to metallic. Our results are relevant for the design of controllable graphene/metal interfaces in functional devices.

Interfacial interactions play a key role in tailoring the properties of graphene and other surface-supported two-dimensional (2D) materials. Remarkably, the effect of such interactions is not limited to the film, but extends to the structures in contact with it, e.g. adsorbed atoms^1^,^2^ and molecules^3^,^4^. For instance, substrate interactions were recently shown to affect the magnetic order of organic monolayers supported on graphene^5^, opening the possibility to add magnetic functionality to this unique material. The study of substrate and interfacial coupling is therefore a technologically very relevant topic, with applications ranging from high performance transistors to spintronics^6^,^7^,^8^,^9^. From the perspective of fundamental studies, a model system permitting to reversibly modify the graphene-substrate coupling is still missing and thus highly desired, as it would enable characterization of the effects of film-substrate interactions on graphene’s properties.

Nickel is an appealing choice in the attempt to engineer such a model system, as it is one of the most technologically relevant substrates for graphene production. Among transition metals, it has a low-cost and is widely available; further, it can readily sustain graphene growth by chemical vapour deposition (CVD), with the additional advantage of requiring lower temperatures than other catalysts^10^,^11^,^12^. Since the lattice mismatch between graphene and Ni(111) is small (~1%), graphene growth occurs preferentially in the top-fcc geometry, maintaining the same orientation as the substrate^13^,^14^. As shown by several studies, rotated graphene phases can also be observed on Ni, with relative coverage controlled by a suitable choice of the CVD parameters and substrate pre-treatment^15^. From an application-oriented perspective, a ferromagnetic support opens up possibilities of spin-dependent transport phenomena^16^. In the graphene-nickel system, however, the semi-metal behaviour of graphene is hindered due to the chemisorption to the substrate^17^,^18^,^19^. Several approaches have been devised to overcome this limitation, in particular by interposing buffer layers that ensure decoupling^20^,^21^,^22^. Since such decoupling is typically obtained chemically or by intercalation techniques, it is an irreversible process^6^,^20^,^23^.

A striking feature of the nickel-graphene system is the formation of interfacial nickel carbides, which segregate from bulk Ni upon cooling from growth to room temperature (RT) and form exclusively below rotated graphene domains^15^. In the present work, we demonstrate the possibility of reversibly decoupling graphene and the substrate “from below”, i.e. by inducing the formation / dissolution of such carbides through the control of temperature. To do this, we investigate at the microscopic level the three possible graphene phases on Ni(111), i.e. epitaxial graphene (hereafter named EG), rotated graphene (RG), rotated graphene with carbide underneath (RGC), and the conversion process between the latter two. In our experiments, we fully exploit an experimental approach combining structure-sensitive low energy electron microscopy and diffraction, with energy-filtered x-ray photoemission electron microscopy and microprobe angle-resolved photoemission spectroscopy, which we use to characterize the local stoichiometry and electronic structure, respectively.

## Results and Discussion

To optimize the relative coverage and the size of the rotational domains, we grew graphene on clean Ni(111) by exposing the sample to ethylene at 540–610  °C. Subsequently, the sample was cooled to RT with a cooling rate of about 1 K/s. Following this procedure, graphene displays coexisting EG, RG and RGC domains. Our microprobe low energy electron diffraction (μ-LEED) data show that in micron-sized RG domains, the lattice is preferentially oriented ± 17° from one of the Ni main crystallographic directions, even though domains with different rotation angles are also observed. A statistical analysis of the distribution of rotational orientation of RG regions can be found in the Supplementary Information online. In the following, we will focus on RG domains with 17° orientation but we stress that the behaviour described hereafter is intrinsic to the RG phase, regardless of the specific rotation angle (see Supplementary Information online).

**Figure 1:**
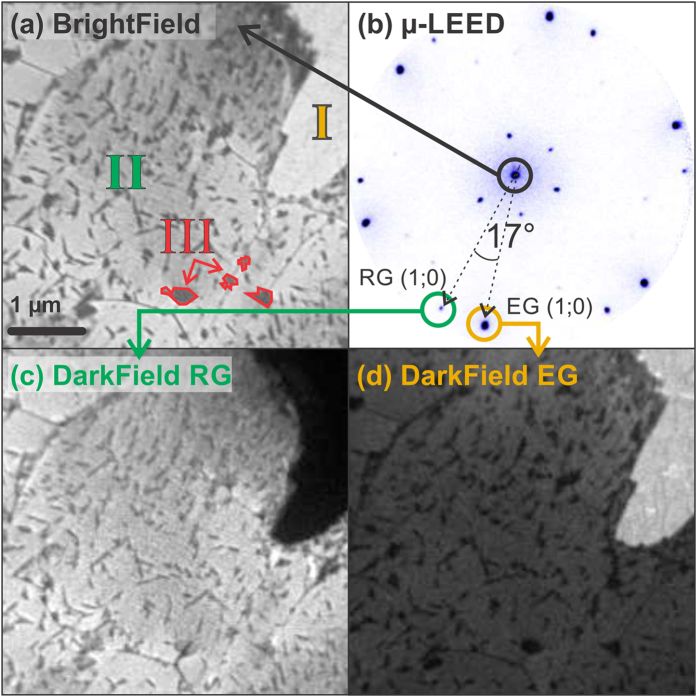
Co-existing graphene domains at the mesoscale. (**a**) BF-LEEM image at V_start_ = 12 V; three different graphene phases, appearing in light, neutral and dark grey, can be distinguished as regions I, II and III, respectively. A few typical patches of region III are highlighted by red contours. (**b**) μ-LEED on the same surface, V_start_ = 55 V; coexisting epitaxial and 17° rotated graphene spots are indicated. (**c**) DF-LEEM obtained using one of the 17° rotated spots in (**b**), V_start_ = 50 V. (**d**) DF-LEEM using one of the spots aligned with the Ni(111) lattice directions in (**b**), V_start_ = 50 V.

The brightfield (BF) LEEM image in Fig. 1(a) illustrates the typical mesoscale morphology of the graphene layer at RT. As the primary diffracted beam is employed, here the image contrast does not distinguish between equivalent rotational domains. Analysis of the image intensity reveals the presence in the film of three coexisting phases, distinguished by different grey levels and labelled I, II and III respectively. Characteristic electron reflectivity (LEEM I-V curve) of each phase is shown in the Supplementary Information. Notably, laterally averaged LEED data over the whole area (see Fig. 1b) show only two patterns corresponding to EG and RG, which cannot be readily assigned to the three distinct regions in the real-space images. The spots closely surrounding the (00) beam are ascribed to double scattering processes between the Ni and RG lattices. Similar diffraction features are also found around the first order RG spots, but become clearly visible only at higher electron energies.

To resolve each phase in real space, we imaged the film using darkfield (DF) LEEM, a method that uses secondary diffracted beams to map the lateral extent of a given surface phase. As shown in Fig. 1(c,d), the images obtained using the first-order diffraction of epitaxial and rotated graphene readily identify region I (II) with EG (17° RG).

**Figure 2:**
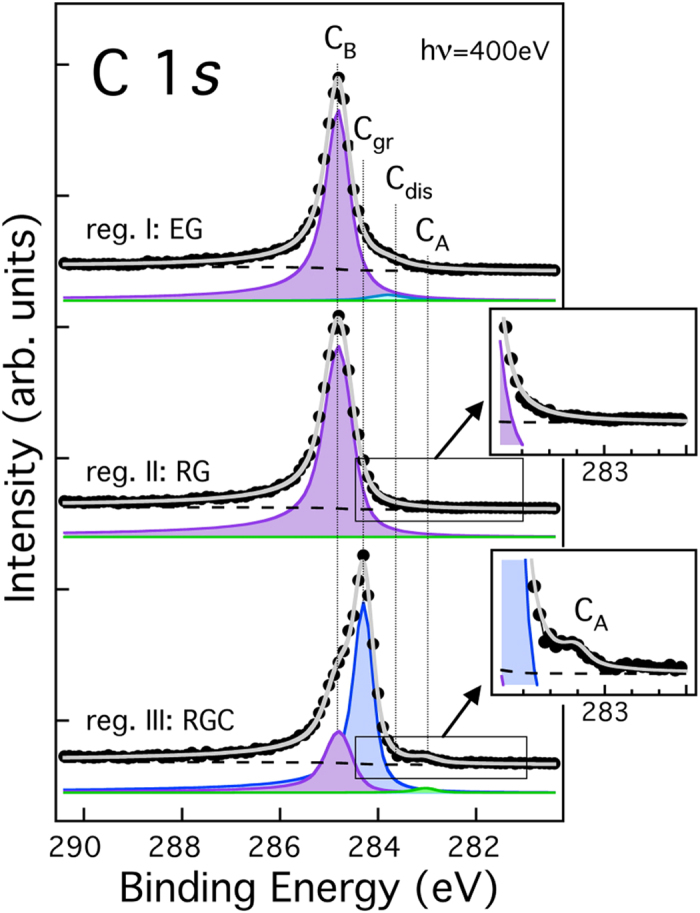
C 1s core level spectra of the different graphene phases extracted from laterally resolved XPEEM measurements (hν = 400eV). Top panel: region I, corresponding to EG phase; mid Panel: region II, corresponding to RG phase; bottom panel: region III, corresponding to RGC phase.

The identification of the small patches labelled III is less straightforward. Their localization within the RG phase, small lateral size ( ≤ 250 nm) and irregular shape suggest that they correspond to RGC areas, as previously imaged by STM^15^,^24^. To verify this hypothesis, we performed laterally resolved X-ray photoemission electron microscopy (XPEEM) measurements on the same region (see Supplementary Information for further details). C 1s core level spectra extracted from a sequence of XPEEM images as a function of photoelectron energy are shown in Fig. 2. Here, each data point represents the average image intensity within well-defined areas located inside regions I, II, and III (top, central and bottom panel, respectively). Data were fitted according to the procedure described in ref. 15, using four components which were attributed to specific carbon species, as follows: C_A_ (green component, 283.2 eV) - surface nickel carbide; C_dis_ (light blue component, 283.8 eV) - interstitial carbon dissolved into the near surface Ni layers; C_gr_ (blue component, 284.4 eV, i.e. the same energy as for HOPG graphite) - weakly-interacting graphene, identified with the RG domains; and C_B_ (purple, 284.8 eV) - epitaxial graphene, used as binding energy scale reference. We note that both components originating from below graphene (C_dis_ and C_A_) have very low intensity, limited by the low effective photoelectron attenuation length (~4.4 Å) at the kinetic energy used^25^. Surprisingly, the C 1s spectrum of both the EG and RG phases is fitted by a single peak corresponding to C_B_, thus indicating that carbon has a similar interaction with the substrate in the two cases. The apparent larger width of the C_B_ peak (~28%) in RG regions is to be expected, considering that, while only two distinct sites contribute to the EG phase^14^, a larger number of adsorption sites is involved when the epitaxial match is lost due to rotation. Conversely, the spectrum extracted from region III is remarkably different. It still shows a spurious C_B_ peak, originating from the adjacent RG regions, which contribute to the spectrum due to the limited lateral resolution of the microscope. However, the main peak is of C_gr_ type, with a small but evident (see inset) carbide component (C_A_) on the low-binding energy side. Such a component, absent in the spectrum from region II (see inset), confirms that region III corresponds to the RGC phase. Notably, the lower value of the C_Gr_ binding energy with respect to C_B_ is typical of systems characterized by a weak interaction between graphene and the metal support^26^.

The present results correct our previous indication of a different binding energy for C atoms in the EG and RG phases^15^. This inconsistency can be explained considering that our previous experiments were performed at RT with a conventional X-ray source in a laboratory set-up, thus integrating over large areas (~ 30 mm^2^) comprising domains of both RG and RGC phases.

**Figure 3:**
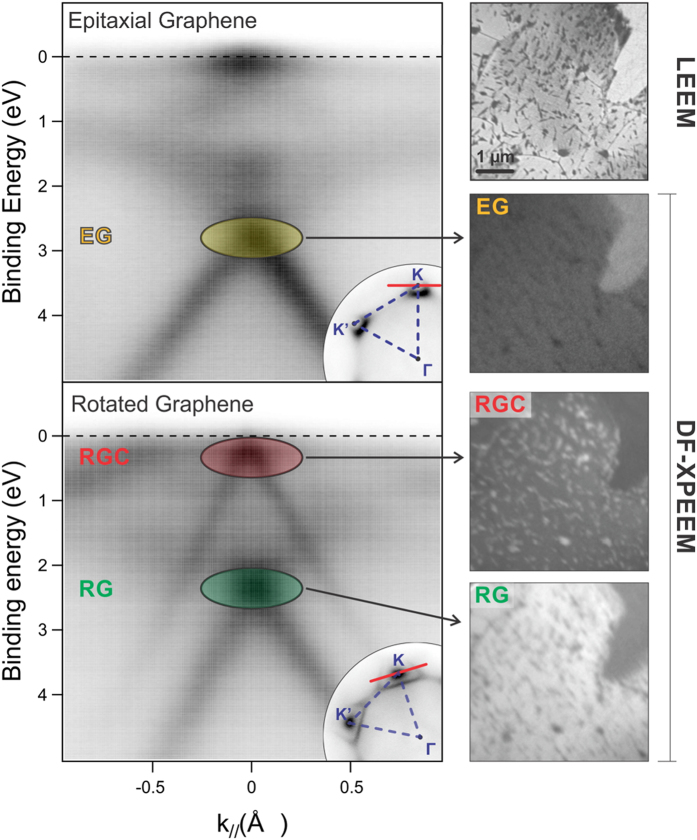
μ-ARPES measurements on epitaxial and rotated graphene regions. Left: momentum distribution curves; hν = 40 eV. The corresponding angular distributions of photoelectrons are shown in the insets acquired at E_B_ = 3.56 eV and E_B_ = 2.58 eV for epitaxial and rotated graphene regions, respectively. Right: Investigated graphene area as imaged by LEEM (top, V_start_ = 12 V), and by DF-XPEEM. The dark-field images were acquired positioning an aperture at the K point in the diffraction plane, at binding energies corresponding to highlighted structures in the MDC curves on the left.

The π band dispersion of the three different graphene phases was investigated by microprobe angle-resolved photoelectron spectroscopy (μ-ARPES). Since our data are collected from a region measuring about 2 microns in diameter, the contributions of the RG or RGC phases in the rotated regions cannot be separated. In Fig. 3 (left), we plot momentum distribution curves (MDC) through one of the K points along a plane normal to Γ-K (see insets) for EG (left top) and mixed RG + RGC (left bottom). In EG regions we find the typical dispersion already reported in literature^19^,^27^,^28^, with a single main feature exhibiting linear dispersion crossing 2.66 ± 0.02 eV below the Fermi energy E_F_, plus a number of minor features at lower binding energies, already attributed to nickel and hybrid graphene-nickel states^7^,^17^. The nature of the strongest structure is presently under debate: it was previously assigned to the presence of a band-gap induced by the interaction with the substrate, but this feature might alternatively be related to a Dirac cone shifted away from E_F_, as suggested by experiments performed at 40 K^27^, or, as proposed more recently, to the main part of a fragmented Dirac cone^29^.

In the MDC from mixed RG and RGC regions (Fig. 3 left bottom), two evident structures show up. One of them (marked in green) closely resembles the main structure found for EG but is shifted about 0.45 eV towards lower binding energy (i.e. 2.20 ± 0.06 eV); the other structure (marked in red) is centred very close to the Fermi level (0.19 ± 0.11 eV), indicating the presence of areas with almost zero doping, and thus of quasi-free-standing nature. To correctly correlate the MDC features with the different phases, we used the darkfield XPEEM (DF-XPEEM) method^30^, using photoelectrons emitted from graphene’s π band at the reciprocal space K point, close to E_F_, to image the surface. In the micrographs on the right hand side of Fig. 3, obtained using this method for each of the observed features, the image intensity is proportional to the local density of states in the film. These images confirm that the MDC single structure at 2.66 eV (yellow) stems from surface areas covered by EG, and allow a clear identification of the two MDC structures recorded from the mixed RG + RGC region to be established. The lower energy structure (green), similar to the EG one, is thus assigned to areas of RG phase. The cone closer to the Fermi level (red) is instead strictly related to the RGC phase (as demonstrated by the inversion of contrast between bright and dark features) and indicates that the small RGC patches are electronically decoupled from the substrate. We note that the 0.45 eV shift in the MDCs from EG and RG phases cannot be due to a different charge state, which would imply a different Fermi energy (i.e. a different C 1s binding energy) not observed in our spectra (see above). Instead, we attribute it to a different overlap of graphene and metal electronic orbitals that leads to different hybridization of graphene π and Ni 3d bands for the two configurations.

**Figure 4:**
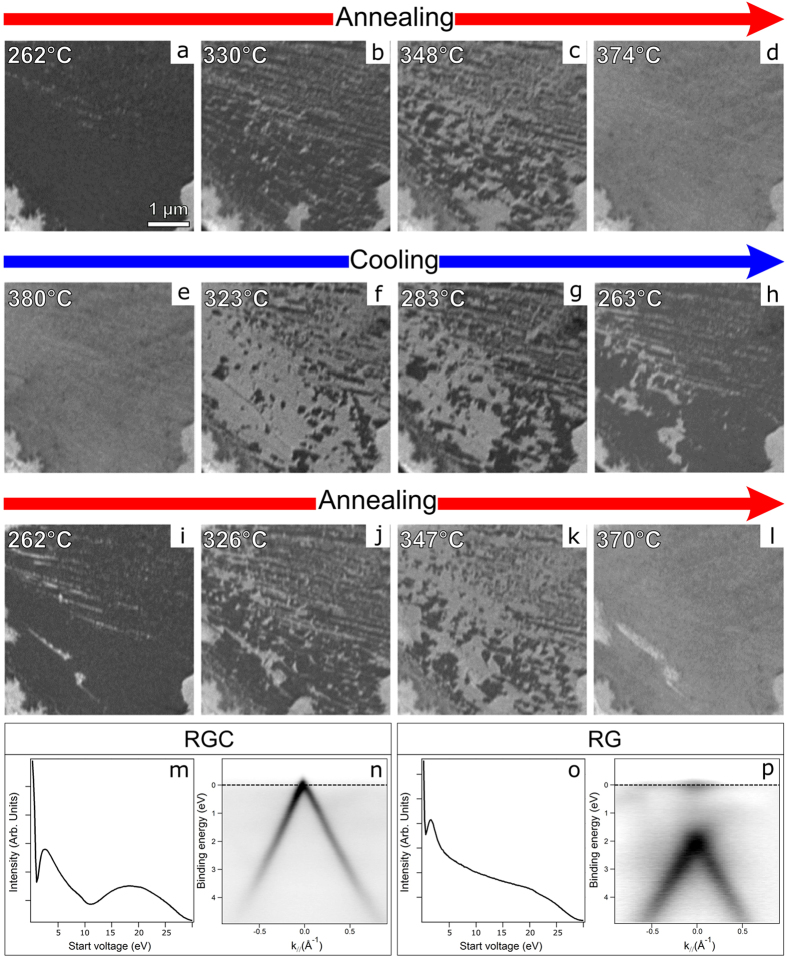
Rotated graphene/substrate coupling and decoupling during temperature cycling. The dissolution of carbide underneath graphene (**a–d**) as the temperature is increased and its re-formation upon cooling (**e-h**) are evidenced by the change in brightness seen in the LEEM sequence (dark = RGC, bright = RG). The epitaxial domains in the bottom corners, which do not change with temperature, are used as markers. A second annealing (**i–l**) converts RGC in RG again. V_start_ = 9 V. (m) I-V curve acquired at RT from the region imaged in (**a**). (n) MDC acquired at RT. (**o**) I–V curve acquired at 370 °C. (**p**) MDC acquired at 360 °C.

The question now arises whether it is possible to control the population of the RGC phase in order to obtain a uniform carbidic buffer and whether this process is reversible. Clearly, such a capability would allow a drastic change in the electronic properties of graphene to be obtained and finely controlled. As shown in our previous work, the RGC phase is not present during growth, but forms only upon cooling towards RT^18^. More precisely, it nucleates at temperatures between 220 °C and 320 °C (extreme values depending on the concentration of subsurface C). Arguably, the amount of time spent in this T range is the key to determine the size of the carbide patches underneath rotated graphene domains. On the basis of this assumption, it is possible to devise a strategy to decouple larger graphene flakes from the substrate. Indeed, the first frame shown in Fig. 4 (panel a) shows a homogeneous RGC region, exhibiting a lateral size of a few microns, obtained after keeping the sample at ~260 °C for few hours. The presence of a uniform carbide layer underneath rotated graphene is confirmed by the characteristic I-V curve (panel m, see Supplementary Information for comparison) and the μ-probe ARPES MDC (panel n), showing a single Dirac cone at the Fermi level (E_D_ = E_F_ – E_kin_ = 0.01 ± 0.06 eV). The Dirac energy E_D_ was determined after calculating the intersection point of lines fitting the two branches of the π band, calculated in an energy interval extending 2 eV below E_F_. These lines were obtained after determining the exact position of each point of the π band branches in the (k_//_,E_B_) space. The error bar on E_D_ (95% confidence level) is a consequence of the statistical errors on the intercept and slope of the lines, not of the limited energy resolution of the SPELEEM.

Upon annealing (panels a to d, complete LEEM movies provided as Supplementary Videos S1, S2, S3 and S4), the carbide dissolves into the bulk and conversion of the initial RGC phase takes place progressively, as revealed by the brightness change in the sequence, until a homogeneous RG is obtained, as confirmed by the corresponding I-V curve (panel o, see Supplementary Information for comparison) and by the MDC (panel p), showing that at ~360 °C the cone is shifted far away from the Fermi level (–2.13 ± 0.08 eV).

The process appears to be entirely reversible, i.e. the carbide inter-layer forms again when the temperature is lowered (panels e-h), dissolves once more when it is raised (panels i-l), and so on. It is therefore possible to reversibly switch between the decoupled/coupled states by simply changing the temperature. We note that the switch between RGC and RG takes place only in the (220 °C–370 °C) temperature range. The carbide coverage remains constant upon further cooling below this range (not shown), while annealing in excess may lead to nucleation of bilayer graphene, as occurs for the thin bright stripe in panel (l). The fact that the conversion is active only in a narrow temperature range implies that rapid cooling of the sample from growth to room temperature yields an almost completely carbide free graphene layer. The temperature range where the switch is active is in agreement with previous investigations on the kinetics of C segregation on Ni samples. Indeed, in pioneering experiments, Blakely *et al*.^31^ analysed the temperature dependence of C segregation on the Ni(100) surface, finding it to be maximum at about 357 °C.

The microprobe ARPES MDCs for the homogeneous RGC and RG phases (panels n and p) provide unequivocal evidence of the effect of the presence of the surface carbide: while direct strong interaction of graphene with Ni induces the formation of hybrid states and a pronounced modification of its electronic structure, carbide formation underneath the graphene sheet decouples microscopic regions of the layer from the substrate^24^, (almost) completely restoring its semi-metal nature. Remarkably, on Ni(111) the *same* rotated regions exist in two states: either decoupled or coupled to the substrate.

Finally, we have measured at high temperature the MDCs of the EG and RG phases (see Supplementary Information), observing only a small shift in the position of the cones with respect to the corresponding phases at lower temperature. This further confirms that the major changes in the electronic structure are indeed due to the presence of interfacial carbide.

In summary, a thorough investigation of the electronic structure of graphene grown on Ni(111) and of its temperature dependence allowed us to devise a strategy to control the graphene-metal coupling. If a surface carbide buffer forms above the topmost Ni layer underneath rotated graphene, the 2D carbon network decouples from the substrate and its semi-metal nature is almost completely restored. Clear evidence is provided that micro-scale carbide domains can be reversibly formed and dissolved by just changing the temperature and deciding the time spent by the sample in the narrow temperature range where the conversion is active. A simple way to vary the graphene/substrate coupling is therefore provided, with significant implications for functional device fabrication and spintronics in particular. The lateral dimension of the switchable regions is only limited by the size of the rotated domains. Systematic studies are ongoing to explore the growth parameter space with the aim of obtaining rotated domains on the millimetre scale.

## Methods

### Sample preparation

The Ni(111) surface was cleaned with Ar^ + ^sputtering at 2 keV and annealing at 600 °C. The cleaning cycles were stopped when the sample did not show any C structure at RT in LEEM and μ-LEED measurements. Growth studies were performed *in-situ* by maintaining the sample at 540-610 °C while back-filling the chamber with C_2_H_4_ (p = 3∙10^−6^ mbar). The surface evolution during growth was monitored with LEEM.

### XPEEM and LEEM Measurements

The photoemission microscopy measurements were carried out using the spectroscopic photoemission and low energy electron microscope (SPELEEM) at the Nanospectroscopy beamline of the Elettra synchrotron.^32^. This instrument combines low energy electron microscopy^33^ with energy filtered X-ray photoemission microscopy^34^. In the SPELEEM, the electron kinetic energy is controlled by a biasing potential on the sample. This bias is referred to as start voltage, V_start_. The kinetic energy of the electrons scattered (or emitted) by the sample is equal to E_kin_ = eV_start_ – δW_i-s_, the latter being the work function difference between the instrument and the specimen. In our measurements, LEEM was used in both bright and dark-field modes, utilizing, respectively, the zero or first order diffraction beam for imaging. The microscope lateral resolution approaches a few tens of nanometres in XPEEM mode, 9 nm in LEEM; the energy resolution is better than 0.3 eV. Besides imaging, the SPELEEM allows diffraction operation mode. Depending whether the beamline photons or low energy electrons are used as probe, μ-ARPES or LEED measurements can also be carried out. The probed area is defined by inserting a field limiting or illumination aperture in the first image plane along the optical path of the instrument. A 2 μm diameter field limiting aperture was used for μ-ARPES, while for μ-LEED three different illumination apertures were employed (500 nm, 1 μm, 5 μm). Part of the ARPES measurements was performed using the darkfield PEEM method^30^ with electrons photoemitted only in a selected region of reciprocal space. In the LEEM experiments, the sample temperature was measured with a C-type thermocouple and checked with an optical pyrometer.

## Additional Information

**How to cite this article**: Africh, C. *et al*. Switchable graphene-substrate coupling through formation/dissolution of an intercalated Ni- carbide layer. *Sci. Rep.* **6**, 19734; doi: 10.1038/srep19734 (2016).

## Supplementary Material

Supplementary Information

Supplementary Video S1

Supplementary Video S2

Supplementary Video S3

Supplementary Video S4
