# Supplementary material for: Switchable graphene-substrate coupling through formation/dissolution of an intercalated Ni-carbide layer
Source: Sci Rep. 2016 Jan 25;6:19734. doi: 10.1038/srep19734 (PMC4726223; doi:10.1038/srep19734)
Supplement: Supplementary Information [file srep19734-s1.pdf]

SUPPLEMENTARY INFORMATION

Switchable graphene-substrate coupling through formation/dissolution-of an intercalated Ni-carbide layer

C. Africh[1,] , C. Cepek[1], L.L. Patera[1,2], G. Zamborlini[2,3], P. Genoni[4], T.O. Menteş[5], A. Sala[5], A. Locatelli[5] and G. Comelli[1,2,5]*

[1]IOM-CNR Laboratorio TASC, Area Science Park, s.s. 14 km 163.5, Basovizza, 34149 Trieste, Italy

[2]Department of Physics, Università degli Studi di Trieste, via Alfonso Valerio 2, 34127 Trieste, Italy

[3]Peter Grünberg Institute (PGI-6), Research Center Jülich, 52425 Jülich, Germany

[4]Department of Physics, Università degli Studi di Milano, Via Celoria 16, 20133 Milano, Italy

[5]Elettra-Sincrotrone Trieste S.C.p.A., s.s. 14 km 163.5, Basovizza, 34149 Trieste, Italy

[*]CORRESPONDING AUTHOR: africh@iom.cnr.it

Table of Contents

1. **I-V curves**
2. **XPEEM images at room temperature**
3. **Rotational orientation of RG domains**
4. **High-temperatures XPS**
5. **μ-ARPES at 450°C**
6. **RG and RGC with -13° rotation**
7. **LEEM movies of switchable coupling**

1. I-V CURVES

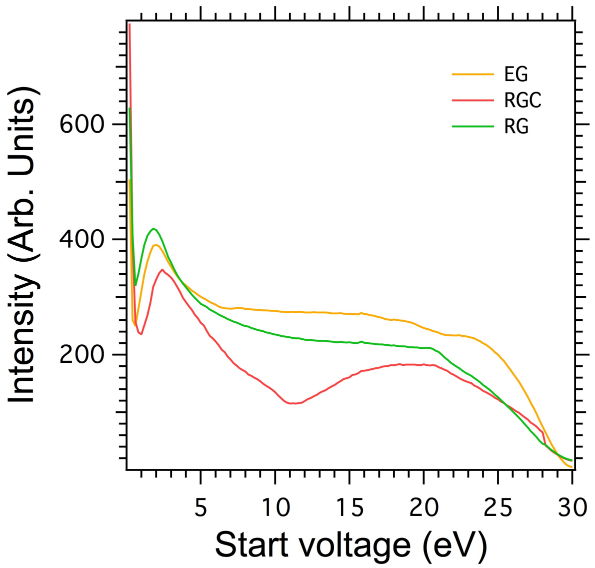

Figure S1: I-V curves acquired on EG, RG and RGC domains.

Characteristic I-V curves (Fig. S1) were acquired by collecting LEEM images of the same surface area at various start voltages and extracting the local intensity (reflectivity) as a function of V_{start} on imaged regions of different phases. EG and RG exhibit similar smooth curves, though can be distinguished by the intensity crossing at ~3.5eV, which implies relative brightness inversion. The RGC phase is instead clearly characterized by an evident dip at ~11eV.

2. XPEEM IMAGES AT ROOM TEMPERATURE

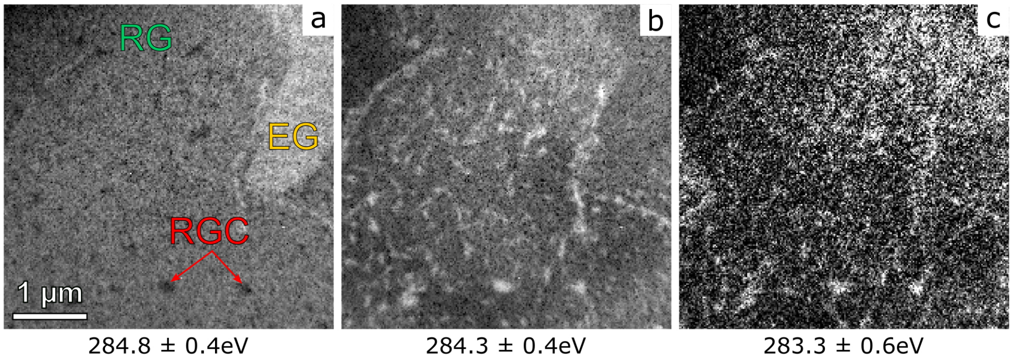

Figure S2: XPEEM images comprising EG, RG and RGC domains. The corresponding binding energies are indicated in the labels on the bottom.

Figure S2 shows XPEEM images acquired at selected energies on a mixed EG/RG/RGC area of the surface. The complete data set was used to extract C1s spectra discussed in the main text and therein shown in Fig. 2. Here, panel (a) corroborates the assignment of C_B (284.8 eV) to EG and RG, while panel (b) confirms the identification of C_{gr} (284.4 eV) with carbon atoms in RGC. Finally, panel (c) correlates the presence of carbide (C_A peak, 283.2 eV) with RGC regions. Note that the poor statistics in panel (c) are due to the very weak photoemission feature imaged.

3. ROTATIONAL ORIENTATION OF RG DOMAINS

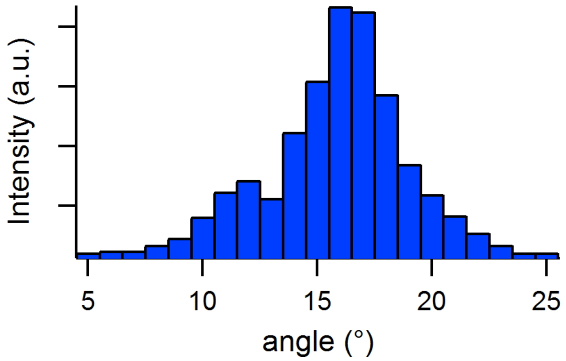

Figure S3: Distribution of rotational orientation of RG domains. The angle is measured with respect to the Ni(111) lattice.

We acquired micro-LEED patterns from 69 different graphene flakes with lateral size larger than 0.5 μm. The statistical distribution of their rotational orientation with respect to the substrate is shown in Fig. S3. Due to symmetry reasons, we plot the absolute value of the rotation angles.

As can be seen, beside the peak at 0° related to EG domains, there is a clear predominance of 17° rotated domains as well as a second peak at 13°. This is not in contradiction with our previous paper, where in a macro-LEED pattern we observed arches of extra-spots approximately centred at ±20°. Indeed, to obtain a more precise comparison, we extracted the intensity profile (not shown) along the arches reported in our old paper, finding a peak centred at ±17° with a FWHM of ~5°, compatible with the present micro-LEED results.

4. HIGH-TEMPERATURE XPS

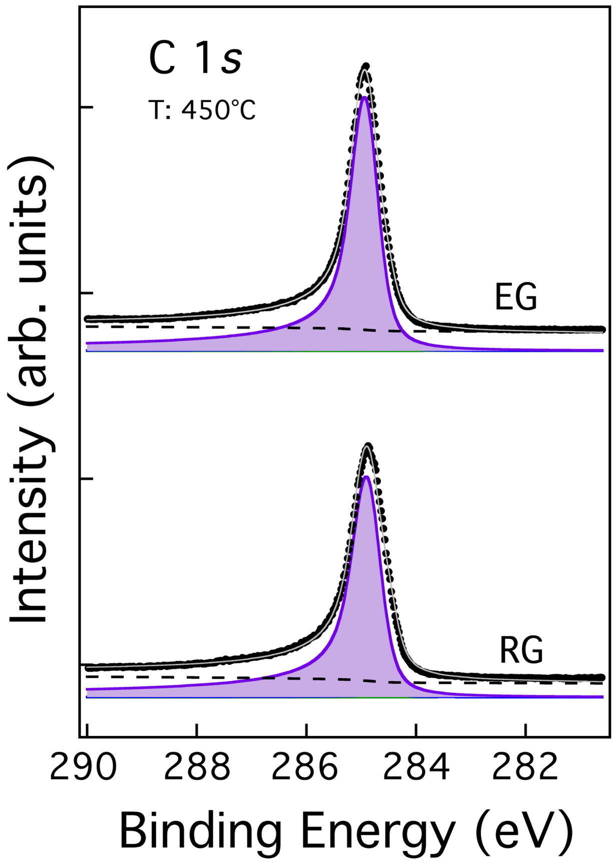

Figure S4: High–resolution XPS spectra measured at 450° C on EG and RG domains (hν = 400 eV).

We measured high-resolution XPS spectra at 450°C on single rotated graphene domains by imaging the dispersive plane of the electron energy filter with field of view of ~2μm (see methods for more details). As shown in Fig. S4, at this high temperature C 1s XPS spectra acquired on both EG and RG regions exhibit a single peak at 284.81±0.05 eV (where the error includes both the fit and the instrumental contributions), confirming the absence of carbide between graphene and nickel, and a similar interaction with the metal substrate.

5. μ-ARPES AT 450°C

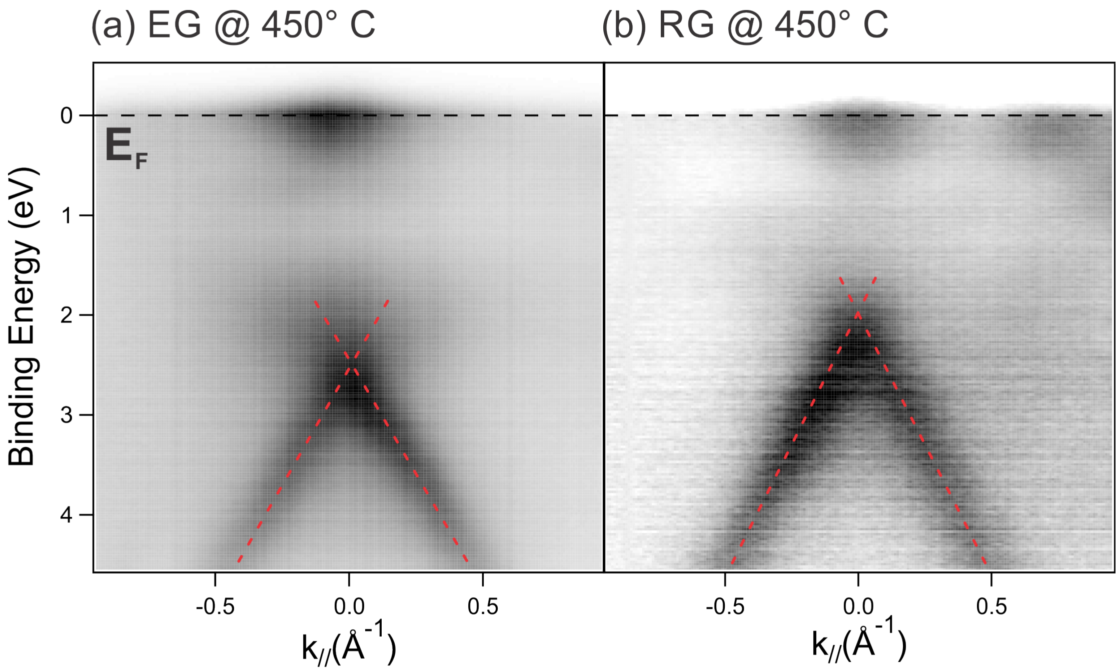

Figure S5: μ-ARPES momentum distribution curves acquired at 450°C on EG and RG domains.

We performed μ-ARPES measurements at 450°C on EG and RG domains. As shown in Fig. S5, on both regions only a single structure was found, centred at E_B = 2.42±0.02 eV and 1.94±0.14 eV respectively. We observe that at this high temperature all band structures, in both EG and RG domains, shift to lower binding energies by ~0.25 eV with respect to RT, which points to a weaker graphene-nickel interaction, possibly related to a lifting of the graphene layer.

6. RG AND RGC WITH -13° ROTATION

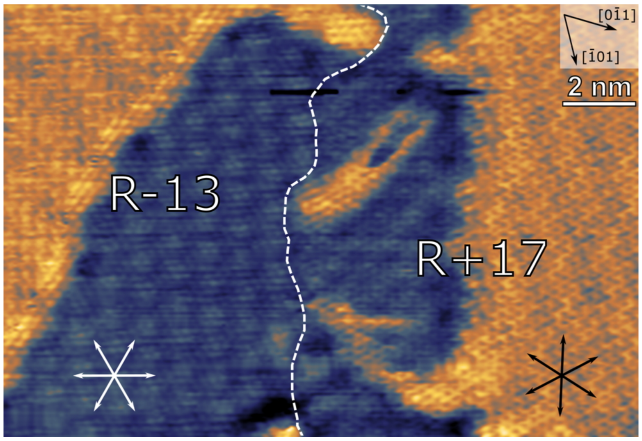

Figure S6: Coexistence of RG and RGC domains with -13° and 17° rotation. STM image at the boundary between graphene domains oriented -13° and 17° off the Ni(111) main crystallographic directions (indicated on the top right corner). The dashed line marks the boundary.

Figure S6 presents an STM image acquired on an area where regions of rotated graphene with different rotation angle coexist, as evidenced by the different moiré patterns of the RG phase (blue areas). In particular, rotated graphene is oriented -13° / +17° off the Ni(111) main crystallographic directions on the left / right side of the boundary, respectively. For both orientations also RGC domains (brighter areas) are present, thus confirming the general validity of the results discussed in the main text, not limited to the specific 17° rotation.

7. LEEM MOVIES OF SWITCHABLE COUPLING

LEEM movies illustrating the reversible switching between decoupled-coupled states are available. All movies have been created by collating LEEM images acquired on the same region of the surface at V_{start} = 10.5 eV. We image a rotated graphene domain initially decoupled from the substrate by a carbide layer (i.e. RGC phase).

Video S1 online illustrates carbide dissolution, and thus progressive graphene coupling to the substrate, during annealing from 260°C to 375°C.

Video S2 online shows carbide nucleation upon cooling to 280°C.

Video S3 online illustrates further carbide growth while keeping the sample at ~280°C, leading to almost complete decoupling from the substrate of the imaged graphene domain.

Video S4 online shows carbide re-dissolution upon new annealing up to 373°C.
